# Etiquette of the antibiotic decision-making process for surgical prophylaxis in Ethiopia: a triangulated ethnographic study

**DOI:** 10.3389/fpubh.2023.1251692

**Published:** 2023-12-18

**Authors:** Getachew Alemkere, Gobezie T. Tegegne, Getnet Abebe Molla, Alemu Belayneh, Hanan Muzeyin, Wendwosen Shewarega, Yidnekachew Degefaw, Addisu Melkie, Workineh Getahun, Hailu Tadeg, Abebe Alemayehu, Eshetu Girma, Wondwossen Amogne

**Affiliations:** ^1^Department of Pharmacology and Clinical Pharmacy, School of Pharmacy, College of Health Sciences, Addis Ababa University, Addis Ababa, Ethiopia; ^2^USAID Medicines, Technologies, and Pharmaceutical Services Program, Management Sciences for Health, Addis Ababa, Ethiopia; ^3^Department of Surgery, School of Medicine, College of Health Sciences, Addis Ababa University, Addis Ababa, Ethiopia; ^4^Antimicrobial Resistance Prevention and Control Case Team, Pharmaceuticals and Medical Devices Lead Executive Office, Ministry of Health, Addis Ababa, Ethiopia; ^5^Division of Nephrology, Department of Internal Medicine, College of Health Sciences, Addis Ababa University, Addis Ababa, Ethiopia; ^6^Department of Preventive Medicine, School of Public Health, College of Health Sciences, Addis Ababa University, Addis Ababa, Ethiopia; ^7^Department of Internal Medicine, School of Medicine, College of Health Sciences, Addis Ababa University, Addis Ababa, Ethiopia

**Keywords:** surgical antibiotic prophylaxis, decision-making, custom, qualitative, surgical site infections, Ethiopia

## Abstract

**Background:**

Prophylactic antibiotics reduce surgery-associated infections and healthcare costs. While quantitative methods have been widely used to evaluate antibiotic use practices in surgical wards, they fall short of fully capturing the intricacies of antibiotic decision-making in these settings. Qualitative methods can bridge this gap by delving into the often-overlooked healthcare customs that shape antibiotic prescribing practices.

**Aim:**

This study aimed to explore the etiquette of the antibiotic decision-making process of surgical prophylaxis antibiotic use at Tikur Anbessa Specialized Hospital (TASH).

**Methods:**

The observational study was carried out at TASH, a teaching and referral hospital in Addis Ababa, Ethiopia, from 26 August 2021 to 1 January 2022. Overall, 21 business ward rounds, 30 medical record reviews, and 11 face-to-face interviews were performed sequentially to triangulate and cross-validate the qualitative observation. The data were collected until saturation. The data were cleaned, coded, summarized, and analyzed using the thematic analysis approach.

**Result:**

Surgical antibiotic prophylaxis (SAP) discussions were infrequent during surgical ward rounds in TASH, leading to practices that deviated from established recommendations. Clear documentation differentiating SAP from other antibiotic uses was also lacking, which contributed to unjustified extended SAP use in the postoperative period. Missed SAP documentation was common for emergency surgeries, as well as initial dose timing and pre-operative metronidazole administration. Importantly, there was no standardized facility guideline or clinical protocol for SAP use. Furthermore, SAP prescriptions were often signed by junior residents and medical interns, and administration was typically handled by anesthesiologists/anesthetists at the operating theater and by nurses in the wards. This suggests a delegation of SAP decision-making from surgeons to senior residents, then to junior residents, and finally to medical interns. Moreover, there was no adequate representation from pharmacy, nursing, and other staff during ward rounds.

**Conclusion:**

Deeply ingrained customs hinder evidence-based SAP decisions, leading to suboptimal practices and increased surgical site infection risks. Engaging SAP care services and implementing antimicrobial stewardship practices could optimize SAP usage and mitigate SSI risks.

## Background

Surgical site infections (SSIs) are the most prevalent healthcare-associated infections in low- and middle-income countries (LMICs), representing a costly and often preventable complication of surgery (1, 2). Antimicrobials are the frequently used medications in surgical wards to prevent and treat SSIs. However, surgical prophylaxis for SSI prevention often involves the improper use of antibiotics in LMICs, contributing to the higher prevalence of SSIs in these regions (3–6).

The high incidence of SSIs in Ethiopia demands immediate attention (7–9). Despite a substantial proportion of broad-spectrum antimicrobials accounting for one-fourth of antimicrobial prescriptions at Tikur Anbessa Specialized Hospital (TASH), SSI rates remained high, exceeding 20% (5, 10). Multiple studies in Ethiopia consistently concluded the prevalence of irrational surgical antibiotic prophylaxis (SAP) use, emphasizing the need for intervention (2, 5, 7–13). These studies highlighted the common occurrences of inappropriate drug choice, initial dose timing, dosing, and duration of surgical antimicrobials, all of which contradict standard treatment guideline recommendations (5, 9–15). A study at Jimma University Referral Hospital in southwest Ethiopia found that 69.3% of antimicrobial use was irrational, resulting in a total direct cost of 2230.15 USD, equivalent to 17.1 USD per patient (9).

The absence of standardized SAP guidelines in tertiary-level hospitals like TASH (16) and unreliable access to essential antibiotics in Ethiopia might also contribute to the problem (17). Additionally, studies reported that surgeons’ resistance is one factor contributing to the irrational antimicrobial surgical prophylaxis use (18). Different qualitative studies have shown disagreement with basic guideline recommendations for SAP use and the enduring influence of culturally ingrained practices (19–21).

Behavioral interventions are recommended to optimize antibiotic prescribing practice (22–26). A study by Broom et al. involving 30 hospitals in Australia found that “sub-optimal antibiotic prescribing is a logical choice within the habitus of the social world of the hospital” (22). Another study in London revealed that emphasis should be placed on addressing prescribing etiquette to influence the antimicrobial prescribing of professionals (24).

Participating in ward rounds is likely the key determinant in shaping collective behavior during therapeutic decision-making (26). It also provides an irreplaceable opportunity to bring together a group of responsible experts to discuss the patients’ cases (27, 28). However, based on the semi-structured interview study of quality markers for clinical wards by Pucher et al., there were problems in surgical ward rounds that may require standardization (29). A ground-up exploration of surgical team functioning, values, and beliefs underpinning surgical antibiotic decision-making in Ethiopian hospitals may also reveal different theories or applications that are important to reform or standardize the practices in the local context. Therefore, this study aims to assess how SAP is prescribed and administered.

## Study methodology

The study was conducted at TASH, Ethiopia’s largest referral and teaching hospital, located in Addis Ababa. The network of TASH staff and the experiences of graduates from the hospital make it a reflection of other healthcare facilities in the region. It has approximately 3,000 healthcare professionals, including 169 specialists. It also has 800 beds, of which 299 beds were dedicated to the surgery department, and 52 different ambulatory clinics, including surgical clinics. Surgical wards have approximately 80 surgeons and 1,200 residents. Recently, TASH has become an antimicrobial stewardship center and study site for hospital-acquired infections in collaboration with the Research Institute of the McGill University Health Center.

The study was carried out in the adult general surgical ward, where surgeons, nurses, anesthesiologists, laboratory technologists, radiologists, and pharmacists provide service. Fellows, residents, and medical interns are also involved in the care process. During the study period, the adult general surgical ward had gastrointestinal and vascular surgery units with 11 surgeons from the upper gastrointestinal, hepatobiliary, colorectal, and vascular subspecialties. The study was conducted between 26 August 2021 and 1 January 2022.

This ethnographic study involved non-participant observations, record reviews, and face-to-face interviews conducted consecutively over 4 months. It aimed to bring an appreciation for and consideration of surgical ward culture and teamwork into a discussion forum for rational antimicrobial use. The study was reviewed and approved by the ethical review board of the School of Pharmacy, College of Health Sciences, Addis Ababa University.

The data were collected by postgraduate clinical pharmacy students who had received training on mixed methods of design, including qualitative and quantitative methods, with a particular focus on ethnographic studies.

### Observation/visits to the ward rounds

Observations were carried out during the first 2 months in the general surgical ward of TASH. Almost all the round observations were carried out after medical interns (undergraduate medical trainees) left for break. The COVID-19 restrictions were also in place during the round observations. The observers visited business ward rounds and other important events. The general surgical ward has vascular and gastrointestinal divisions. The observations were performed in 2 units, C5 and D5, on the fifth floor. The two divisions performed their business rounds independently. We first observed the gastrointestinal (GI) surgery unit rounds, followed by the vascular unit rounds. A printed observation guide was used for the observations. The observation data were typed daily into a Google Form. The observation findings were analyzed, categorized, and synthesized immediately for use as input in consecutive interviews.

### Document review

Surgical antibiotic-related guidelines, protocols, standard operation procedures, patient medical records, and information communication and education materials were targeted for review. All medical records available at the nursing station with an assumed prophylactic antibiotic were reviewed. A review of the national guidelines and pharmacy-related documents was already covered in our previous publication (17). Data were collected using a checklist.

### Interview

A mixed sampling technique was employed, involving convenience sampling where participants were recruited based on proximity, accessibility, and willingness to participate, and snowball techniques. A semi-structured interview guide was developed based on the findings of the ward round observation and medical record review. The interview guide included questions about specific experiences and perspectives on the decision-making process of SAP use, as well as opportunities and challenges for antimicrobial stewardship within surgery. Open-ended questions were used to explore participants’ views, perceptions, and their experiences. Overall, 14 interviews (three women) were scheduled through telephone calls, emails, and face-to-face interactions. Three individuals declined participation: a surgeon due to time constraints, a fellow who suggested contacting surgeons, and a nurse with less than a month of experience in the study wards. In total, 11 healthcare professionals (two women) from the general surgical ward participated in face-to-face interviews: two surgeons, three residents, two anesthesiologists, two nurses, and two intern students. These interviews were conducted in their workplaces. In one interview with a surgeon, a fellow was present. One interview took place at the participant’s home via Google Meet. While nine of the interviews were carried out by two interviewers, the remaining two were carried out by one interviewer. Most of the interviewees (8) have experience in the surgical wards for more than 1 year (exposure ranging from 1 month to 6 years) and had a recent (within 1 year) exposure to antimicrobial resistance (AMR) concepts. The interview ranged from 40 min to 85 min. Audio records were used during the interview, but four interviews refused audio recording. We discontinued further interviews as the data saturation was achieved, as evidenced by the consistency of findings across the ward round observation, medical record review, and interview data. The methods outlined in this work were assessed for adherence to the consolidated criteria for reporting qualitative research (COREQ) checklist (30). A copy of the COREQ checklist can be found in Annex 1.

### Data rigor and analysis

Field notes were entered on the day of the observation to ensure rich and accurate documentation. Interviews were recorded and transcribed verbatim. Data were analyzed using the thematic analysis approach. Observation notes, document analysis, and interview transcripts were coded to identify key concepts and develop them into themes. The lead researcher coded the ward observation and medical record review data, which was then reviewed by the data collectors. Interview data coding involved a discussion between two data collectors, followed by a review by the lead researcher. Final categorization and the theming were determined collaboratively by the lead researcher, data collectors, and other researchers. Double coding was not employed. Data categorization and theme development were performed in a step-by-step manner for each data collection method. Following the first round of observations, findings were analyzed, categorized, and themed. These interim findings informed the subsequent document review. A new theme, derived from the observation and record review findings, was then developed and integrated into the interview guide preparation. Interview findings were subsequently analyzed, categorized, and themed anew based on the theme developed in the previous data collection phase. The coding and analysis were carried out manually. Analytic rigor was enhanced by probing for atypical and contradicting cases during coding and theme development. Inter-rater reliability was ensured by integrating all the research team members in the final analysis. These different approaches qualify triangulation and cross-validation of the findings.

Finally, all the data (transcripts, notes, and coding reports) were retained and added to the documentation of research aims, design and sampling, and recruitment processes and practices to form an audit trail.

## Results

Overall, 21 round observations, 30 chart reviews, and 11 interviews were conducted consecutively over 4 months to observe the etiquette of the SAP decision-making process. The results are presented as follows:

### Ward round observations

Overall, 21 ward rounds, totaling 1,069 min, were observed. These included 14 gastrointestinal (GI) surgery and 7 vascular surgery team rounds. The ward rounds typically began after 09:00 AM each day but sometimes began after 10:20 AM. The round times ranged from 20 min to 1.3 h. Some fast rounds were conducted for a limited number of patients, as few as four. However, regular rounds covered all admitted patients occupying available beds. The number of attending teams and the durations of ward rounds varied. The round team consisted of two to six members. Almost all fast rounds were conducted by two or three members for a few patients to make an early decision (surgery preparation, consultation). Female rounding teams were rare (the maximum seen per round is three), and sometimes, the rounding teams were all men. No round was conducted without residents. They were assigned on a monthly rotational basis. Senior surgeons were available upon consultation. Except for students who were rarely seen, nurses and other healthcare professionals were not part of the rounding team. Pharmacy and nursing students participated in the rounds inconsistently (Table 1).

**Table 1:** Summary of the ward round context and attendants’ demographics.

Observation/visit	Duration (minutes)	Number of round participants (Total)	Number of round participants (Female)	Number of Patients served	Led by	Round participants and remarks
1	67	6	0	21	Resident	Residents + students
2	80	3	1	22	Resident	Resident
3	43	3	1	21	Resident	Resident
4	0	0		0	None	
5	50	6	2	25	Surgeon	2 surgeons (one as a consultant for two GI cases +4 residents)
6	70	3	0	26	Resident	1 Surgeon and 2 Residents
7	30	3	1	10	Resident	Residents
8	60	8 (2 are pharmacy students)	2	25	Resident	6 Resident + pharmacy students
9	40	3		4	Resident	Resident
10	65	3	1	16	Resident	Resident + GI consultant (returned after discussing two cases)
11	80	3	1	5	Resident	Residents
12	35	4	1	23	Surgeon	Residents, one Surgeon
13	40	5	1	21	Resident	Resident
14	30	5	2	7	Surgeon	Surgeon, Residents
15	40	3		7	Surgeon	Surgeon, Residents
16	40	4	1	7	Resident	Residents
17	50	5	2	20	Resident	Residents
18	60	6	2	20	Resident	Residents
19	44	9 (3 are pharmacy students)	3	19	Resident	Residents
20	100	5	2	25	Surgeon	Surgeon, Resident
21	45	6	2	5	Fellow	Fellows, medicine, and pharmacy students
	1,069			329		

GI, gastrointestinal.

### Medical record review

Overall, 30 medical records were reviewed. One medical record had clear documentation showing infection, justifying the continued antibiotic use and replacement. A total of 11 of the surgeries were emergency, and 19 were elective. Medical intern students and junior residents were involved in prescribing SAP, but this may not mean they were the ultimate decision-makers. While nurses were usually involved in antibiotic administration in the wards, SAP in the operating room was usually administered by the anesthesiologist/anesthetist. Ceftriaxone, with or without metronidazole, is the default SAP prescription. Ceftriaxone was given pre-operatively and postoperatively, while metronidazole was consistently documented in postoperative records. In emergency surgeries, pre-operative documentation of ceftriaxone was often missed. The wound class and pre-surgery dose timing were often not documented. Prophylactic antibiotics were often given for a prolonged period without evidence of infection. SAP choices, dosing, and duration were often deviant from the current evidence-based recommendations (31, 32). These SAP decisions were not consistently discussed on the ward rounds (Table 2).

**Table 2:** Summary of chart review.

Variable	Category	Frequency (%)	Comment
Age, years (mean ± SD)		44.73 ± 21.11	
Sex	Male	21 (70.0)	
	Female	9 (30.0)	
Surgery type	Elective	19 (63.3)	
	Emergency	11 (36.7)	
Wound class	Contaminated	1 (3.3)	
	Unspecified	29 (96.7)	
Class of surgery	Gastrointestinal	15 (50.0)	
	Vascular	11 (36.7)	
Duration of operation, minutes (mean ± SD)		169.9 ± 106.2	
Prescriber of antibiotics	Medical Intern	17 (56.7)	The prescriber is identified by the signature on the medical record, but they may not be the ultimate decision-makers. Antibiotics are administered by nurses. A resident said that “SAP before 30 min is usually given by anesthesiologist, sometimes by nurses on call”
	Resident	13 (43.3)	
Antibiotic	Ceftriaxone	13 (43.3)	Metronidazole’s pre-operative use is undocumented, while postoperative use is consistently recorded. One patient’s antibiotic regimen was changed from ceftriaxone to vancomycin, ceftazidime, and metronidazole.
	Ceftriaxone and metronidazole	17 (56.7)	
Pre-surgery dose of antibiotics	Inappropriate	29 (96.7)	Except for one instance, ceftriaxone 1 g administration is deemed inappropriate (low) as per guideline dosage recommendations.
	Appropriate	1 (3.3)	
Pre-surgery dose timing of antibiotics	Within 60 min	14 (16.7)	The missed documentations are common for emergency surgeries
	Undocumented	16 (53.3)	
Duration of antibiotics, hours (mean ± SD)		69.2 ± 85.6	The term ‘pre-op order’ often implies prophylactic use, making it unclear whether the antibiotic prescription is for treatment or prevention, especially for administering nurses during the postoperative period. Despite some documentation suggesting that surgeons prefer to continue SAP for more than 24 h due to hygiene concerns, no justification for prolonged SAP use was found. A resident said, *“we give antibiotic prophylaxis for a week for gastrointestinal surgeries…..”*

### Triangulated observation findings

The analysis identified five key themes on antibiotic management in surgical wards: (a) round operation, (b) surgical rounds precedence for SAP decision, (c) communication troubles, (d) etiquette of SAP decision-making (further divided into two sub-themes), and (e) opportunities suggested for improving SAP management.

#### Round operation

Residents and/or medical interns conducted daily rounds and were responsible for looking after their respective patients admitted to their beds. They were the core practitioners who supported the patients and consulted with senior surgeons and other experts across horizontal departments for antibiotic and other medical management. Senior surgeons were highly engaged in operating room activities and were available for consultation during rounds, but they commonly left the rounds after addressing selected cases. Other professionals do not engage in regular rounds. Every morning, before the round started, the nursing staff ran around to administer medications and provide nursing care. Sometimes, nursing care was also provided along with the rounds by a separate nursing team (Table 3, section T1a).

**Table 3:** Themes of the triangulated findings (non-italic notes were collected from all observations; sample interviewee sayings are under the quotation and in italics).

Code	Observations + interview response examples
Theme 1: Round operation	
T1a	[MDT engagement] All rounds are conducted by residents and interns. Surgeons also participate in regular rounds, but mostly upon consultation. Other healthcare professionals, except for pharmacy and nursing students who attend inconsistently, did not participate in the surgical rounds. *“Yes, it is correct. As you observed in this ward, the round is carried out only by the physicians and this will affect the quality of the healthcare service. From my experience in another setting, the involvement of especially clinical pharmacists had improved patient care….”* Interview Resident B.
T1b	[Work dynamics] There was a fluid hierarchy for leading ward rounds, with people promoted or demoted depending on who was present.
Theme 2: Surgical rounds and their precedence for SAP	
T2a	[It is the focus, not the site] SAP was discussed in less than 10% of the round observations, and the discussions were limited to the duration of antibiotic use. Medication-related discussions were generally not central to the surgical team. *“Surgical procedures performed, possible complications and others are well planned and discussed, but SAP [surgical antibiotic prophylaxis]….. Not discussed at all. I didn’t see any communication regarding SAP choice, dose, and related issues during my stay at this ward.”* Interview, Resident A
T2b	[It is the custom, not the site] SAP was usually decided outside the surgical rounds, following default procedures. *“It [surgical antibiotic prophylaxis] is rarely discussed [in the rounds] such as for the sake of teaching-learning. In my opinion, the place where SAP-related issues decisions should be chosen depends on the patient. Like for emergency events, it is better to be decided at OR by the senior surgeon or resident and for elective ones, it can be discussed and decided in the ward when the patient is prepared.”* Interview, resident B
Theme 3: Communication troubles	
T3a	[Missed SAP communication or documentation] Significant omissions were found in SAP administrations and communications. For example, pre-operative antibiotic administration documentation was often missed for emergency surgeries, and metronidazole dosing was not documented pre-operatively for either elective or emergency procedures. “*Based on my experience SAP is given in OR before anesthesia. Most of the time elective surgery has several steps, therefore there will be more documentation in case of elective surgery but in case of emergency surgery there may be poor documentation, but it doesn’t mean the patient did not get pre-OP SAP. In the case of metronidazole, it may be added after surgery if there is suspicion of contamination and anaerobic involvement.”* Interview, Anesthesia A
T3b	[Troubled SAP communication] Although the pre-operation orders state “for 24 hours” SAP may be administered for more than 24 hours. “*In my experience, most of the time prophylaxis is discontinued within 24 hrs. If the patient took it for more than 24hrs in contrary to the plan written on the chart, there might be a problem in communication. …. Especially nowadays there is only a business round, which is very fast, and no other team like the nursing team on the round. So, it might be not discussed to discontinue even if the patient is taking it for extended days.”* Interview, Resident A
T3c	[Is SAP clear for non-prescribers?] It can be difficult for nurses to differentiate the purpose of antibiotic use without updates, especially when ordered in the post-operative period when the “pre-operative order’ label is no longer applicable. “*Sometimes the purpose of antibiotics may not be recorded whether it is for surgical prophylactic or for treatment purpose, but we can understand it by the nature of the case*.” Interview, nurse B “*If it is written as “ceftriaxone for 24hrs” that is also part of prophylaxis, even though the documentation doesn’t say it is for treatment or prophylaxis. So, by default, it’s known*.” Interview, resident A
Theme 4: Etiquette of decision making: Delegated surgical antibiotic management	
T4a	*“Yes, everyone is a decision-maker. Interns can prescribe antibiotics because of the transferred learning and authority to them”* Interview, Senior surgeon A
T4b	*“In our practice, it is difficult to say who decides on SAP, … The prescriber is a surgeon but sometimes the anesthesia team will prescribe….. interns and residents could prescribe SAP even including reserved medication.”* Interview, Anesthesia B
T4c	*“I understand that the person [Resident/Intern] who writes and signs SAP prescription orders might be his/her own decision, unlike other medication orders. Because there is no discussion on SAP and the medication given as SAP is also known.”* Interview, Resident B
T4d	*“The SAP could be documented and signed by the resident or intern on the medical charts but that doesn’t necessarily mean that intern or resident is the decision maker. He/she might document what was decided by the senior physician or another senior resident.”* Interview, Resident C
Theme 5: Etiquette of decision making: Established customs than evidence-based practices	
T5a	[Custom is the guide in the absence of institutional guidelines based on local data] No facility guideline or clinical pathway is available in the general surgical wards. “*I did not know any reference materials, protocols, or guidelines used for SAP decisions in TASH [Tikur Anbessa Specialized Hospital].”* Interview, nurse A “*We use international guidelines due to the absence of local data.”* Interview, Senior Surgeon A
T5b	[Drug unavailability shaping the practice] Drug availability and/or the prescribers knowledge of available medications can shift the prescribing behavior and trends. *Ceftriaxone and metronidazole are widely available medications. For this reason, the physician may prefer to prescribe those medications. Now it is being the trend to use that medication and the trend is by far more difficult to change the practice rather than the non-availability.”* Interview, Anesthesia A *“Drug unavailability and awareness gaps on the available medications is critical”* Interview, senior surgeon A
T5c	[Ceftriaxone is the new normal] For whatever reason, there was a well-established practice of using ceftriaxone indiscriminately for SAP. Additionally, concomitant metronidazole use was reserved when aerobic coverage is required. “*There is no authorization required regarding SAP decision-making because everybody knows that ceftriaxone is given…. As I said early, now it is a trend to use ceftriaxone and it is difficult to change the practice without creating awareness and availing first-line antibiotics.”* Interview, Resident A
T5d	Practitioners believe that their learning and practice differ, but they still follow the practice. *“I think we are not applying what we learned regarding SAP choice based on the type of surgery, even timing of administration, and others.”* Interview, Resident B

*“….the round is carried out only by the physicians and this will affect the quality of the healthcare service….”* Interview, Resident B, Table 3, section T1a.

There appears to be an established hierarchical decision-making process, with decisions passed down from senior to junior team members. However, residents who did not have any obvious hierarchies for new observers led most rounds. The hierarchies became more evident when undergraduate students were present, with the senior leading the discussion, asking questions, and explaining occurrences (Table 3, section T1b).

Residents presented all patient cases. Nurses were also asked for information, usually related to drug administration. In contrast, junior residents documented the round decisions. Except for cases documented directly in the patient chart, most documentation was made either in an exercise book or on a piece of paper. This documentation was transferred to the patient chart and/or to the i-Care (TASH electronic record system) in the nursing station room after the rounds. Some rounds were also conducted with minimal or no documentation during rounds.

#### The surgical round’s precedence for antibiotic prophylaxis decision

Most rounds primarily focused on the surgical procedures, with little attention given to SAP. SAP was discussed in no more than 10% of the observed round sessions (Table 3, section T2a). Routine SAP prescribing was handled in a default manner outside of the ward rounds (Table 3, section T2b).

*“It is rarely discussed [in the ward rounds] such as for the sake of teaching-learning. In my opinion, the place where SAP-related issues decisions should be chosen depends on the patient. Like for emergency events, it is better to be decided at operation room by the senior surgeon or resident and for elective ones, it can be discussed and decided in the ward when the patient is prepared.”* Interview, Resident B, Table 3, section T2b.

#### Communication troubles: documentation versus oral communications

Surgical wards had suboptimal and uncoordinated antimicrobial management communication. Many communication lines existed, such as from surgeon to the resident, senior resident to the junior resident, previous rotation team to newly joining team, physician to nurse, and other horizontal healthcare teams to the surgical care team, either orally or through documentation. However, these communications were poorly coordinated, which increased the likelihood of missing SAP-related information in surgical wards. For example, missed pre-operative SAP administration documentation was common for emergency surgeries, as opposed to elective procedures. Similarly, metronidazole was not documented in the pre-operative period, regardless of the surgery type (Table 3, section T3a).

“*Based on my experience SAP is given in the OR before anesthesia. …. in case of emergency surgery there may be poor documentation, but it does not mean the patient did not get pre-OP SAP. In the case of metronidazole, it may be added after surgery if there is suspicion of contamination and anaerobic involvement.”* Interview, Anesthesia A, Table 3, section T3a.

Additionally, although the common pre-operative order states ‘for 24 h,’ SAP was administered for more than 24 h without a clear justification (Table 3, section T3b). This prolonged postoperative use of SAP made it difficult to differentiate the purpose of the antibiotic use (Table 3, section T3c). Another communication problem was inconsistent documentation seen in the patient’s medical and electronic records.

“*Sometimes the purpose of antibiotics may not be recorded whether it is for surgical prophylactic or for treatment purpose, but we can understand it by the nature of the case*.” Interview, Nurse B, Table 3, section T3c.

#### Etiquette of decision-making: delegating antibiotic management

As the primary practitioners supporting patients and consulting with senior surgeons and other experts from different departments, residents and medical interns were responsible for antibiotic decisions (Table 3, sections T4a and T4c). While senior residents delegated junior residents or interns, they may have been the ones who decided on antibiotics (Table 3, sections T4b and T4c). In contrast, prescription documentation and communication were left to the junior residents and medical interns (Table 3, section T4d).

*“…Interns can prescribe antibiotics because of the transferred learning and authority to them”* Interview, Senior Surgeon A, Table 3, section T4a.

#### Etiquette of decision-making: established customs rather than evidence-based practices

Ethiopia has an AMR national action plan (2021–2025) (33), standard treatment guidelines (34), and an antimicrobial stewardship practical guide (35) that can govern antimicrobial use in hospitals. The 2021 standard treatment guideline for general hospitals includes dedicated sections for SAP (34). However, surgeons in tertiary-level hospitals often disregard these guidelines, as evidenced by the statements of a nurse and a surgeon, who acknowledged the lack of reference materials or guidelines used for SAP decisions at their institution (Table 3, section T5a). Additionally, the surgeon noted that the absence of local data to guide antimicrobial use has led surgeons to rely on international guidelines (Table 3, section T5a). However, a review of medical records revealed that the surgeon’s SAP use practice is not in line with international guidelines either (Table 2).

This disregard for guidelines was further exacerbated by the lack of a facility-based SAP guideline or clinical protocol for SAP (Tables 2, 3 section T5a). Moreover, practitioners’ exposure to available broad-spectrum drugs was more influential than their knowledge of which medication to prescribe according to the guideline recommendations (Table 3, section T5b). For example, despite the national guideline recommendation, cefazolin was not available for a long period (17). Consequently, indiscriminate use of ceftriaxone was seen as the default practice, reflecting the custom-guided practice that prevailed over evidence-based recommendations (Table 3, sections T5a to T5d).

“*There is no authorization required regarding SAP decision-making because everybody knows that ceftriaxone is given….it is a trend to use ceftriaxone and difficult to change the practice without creating awareness and availing first-line antibiotics.”* Interview, Resident A, Table 3, section T5c.

### Opportunities to improve antibiotic management

The problems identified in the etiquette of SAP decision-making suggest six categories of intervention: (a) access to essential antibiotics and a policy on the management of essential antibiotic shortages, (b) multidisciplinary team engagement, (c) promotion of evidence-based practices, (d) antimicrobial stewardship intervention, (e) awareness creation and effective communication, and (f) better education of the surgical staff on antimicrobial use (Table 4).

**Table 4:** Interventions to optimize surgical antibiotic use.

Interventions	Specific actions
Multidisciplinary team engagement	Surgeon leadership Infectious disease consultation Microbiology and pharmacy engagement Effective communication with nursing and anesthesia
Ensuring sustainable essential antibiotic access	Access to essential antibiotics Policies/alternative recommendations in case of essential antibiotic shortage
Evidence-based practices	Availing and adhering to evidence-based local guidelines Data-driven decision-making
Antimicrobial stewardship interventions	Assigning responsible teams National policies, facility-specific clinical protocols Compliance assessment Stop order sheets for postoperation SAP use Differentiating SAP use from other antibiotic uses
Awareness and effective communication	Awareness of the available antibiotics, guidelines, protocols, and interventions Redesigning patient medical records to support optimal antibiotic use Soliciting communications across different care units and the MDT team Effective overhanding of patient history upon rotation and referral
Better education of the surgical staff on antimicrobial use	Infectious disease consultation Infection and antimicrobial resistance specialty education Pre-service training Inservice training addressing major caretakers (including residents and medical interns)

## Discussion

AMR and its spread are a global threat (36). Although it is a global problem, AMR remains a major threat to sub-Saharan African countries like Ethiopia (2, 37). This AMR crisis has been attributed to improper drug use (overuse or misuse), poor antibiotic regulation, limited antimicrobial stewardship, poor prescribing habits, non-compliance with prescriptions, and lack of new drug development by the pharmaceutical industry (36–38). Unregulated antimicrobial use in surgical wards remains a common problem in Ethiopia, especially for surgical prophylaxis (2, 5, 6, 11, 17). The inappropriate use of antimicrobials stems from a multitude of factors, including the unavailability of facility-level guidelines or policies exacerbated by excessive prescriber autonomy and a lack of local data, communication barriers, unstable antimicrobial supplies, inadequate prescriber awareness and knowledge, unclear role and responsibility frameworks among practitioners, and prevailing customs (39). We employed an ethnographic study to explore the potential reasons for the inappropriate use of SAP in Ethiopia.

Ethnographic research is an interesting qualitative approach that involves observation (participant and non-participant), interviews, and textual analysis to gain insights into cultural practices. Although rarely used in our country, ethnography has a long history of significance in healthcare research (40–42). The quality of qualitative ethnographic studies can be determined by the length of observations and the extent of immersion. We conducted a 4-month-long observational study in the general surgical ward of a low-income country hospital to explore the etiquette of SAP management.

**Figure 1:**
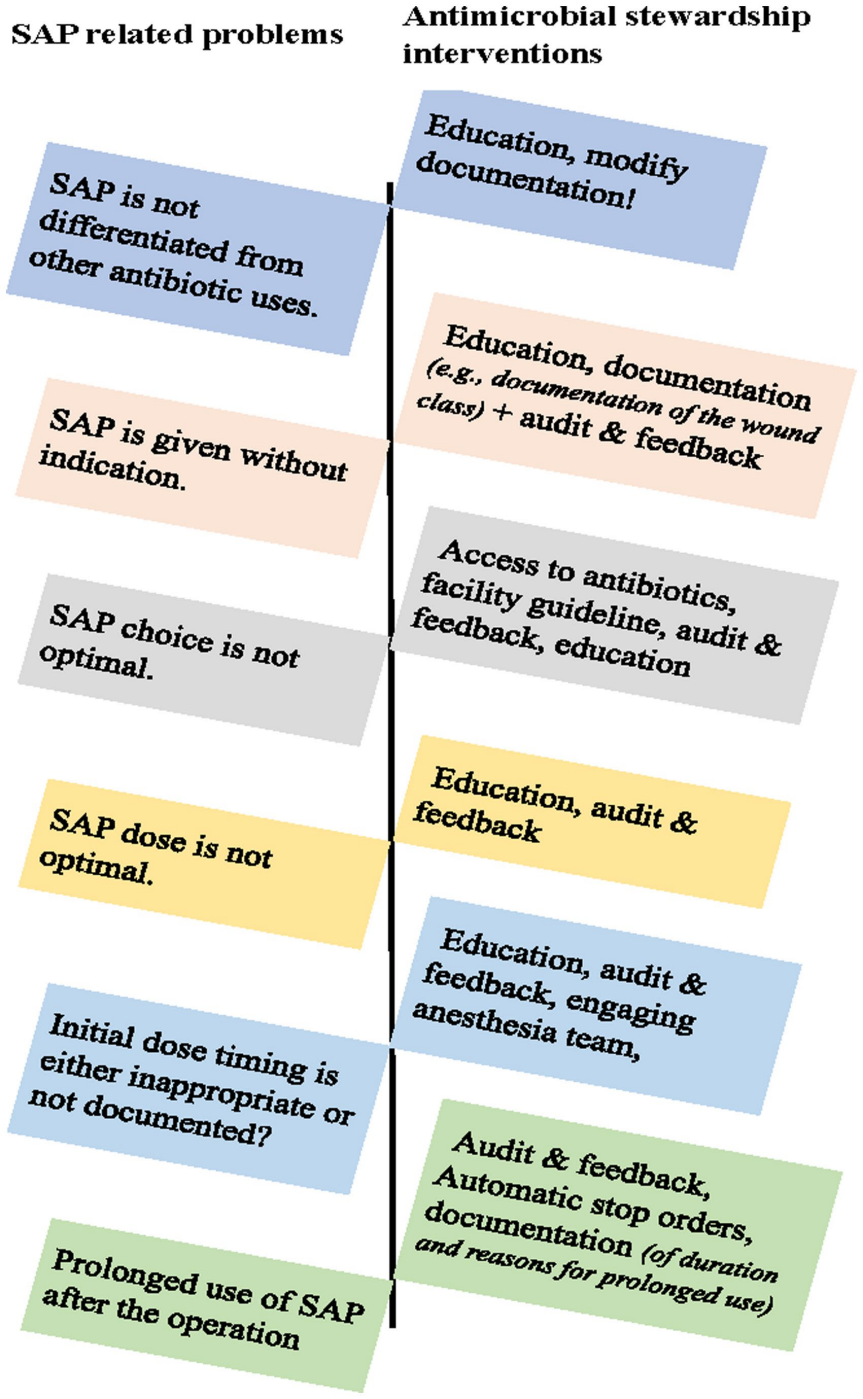
Antimicrobial stewardship interventions to optimize surgical antibiotic use.

The SAP decision-making process was influenced by two paradoxical contexts: the focus of care (Table 3, section T2a) and the custom of care (Table 3, section T2b). This is supported by other published studies (24, 26). Although surgeons bear the major responsibility for patient care, they assume their primary objective is surgical care issues specifically dedicated to the operation room activities and associated consultations (26). Other duties, including antibiotic management, were peripheral to the surgical care team and often missed in the rounds. However, the default custom on SAP decisions plays an equally important role, commonly outside the ward round periods (24). The findings of this study suggest a need for a quality improvement intervention to optimize SAP prescribing (Table 4; Figure 1). Understanding and addressing the determinants of SAP prescribing behaviors are keys to optimizing antimicrobial use and combating AMR.

The surgical pathway is complex, with different actors involved in patient care. Although the multitude of communication pathways and rotations in TASH are necessary, they can introduce gaps in communication that hinder effective coordination and lead to mistreatment in patient care, especially in the absence of binding procedures (26). Critical missed communications were observed in the SAP decisions and management in the surgical wards (Table 3, sections T3a and T3b). Therefore, quality improvement interventions can aim to redesign the patient medical records (Figure 1) to include dedicated sections on SAP use, including mandatory documentation for the identification of prophylaxis from the treatment orders, automatic stop order forms, and compliance assessments to the surgery protocols on the peri-operative antibiotic administration requirements (43).

The lack of priority on SAP decisions and the absence of binding policy/documentation was compounded by the lack of drug expertise, resulting in antibiotic decisions being based more on prevailing custom than evidence recommendations (44). Their antibiotic choice, dosing, and timing practices often go against the guideline recommendations due to several reasons, including the prevailing culture of prescribing. Surgical teams also unnecessarily use antibiotics in the postoperative period due to the fear of infection and long-held practices (17).

Major interventions to optimize SAP use should also target residents and medical interns, as they are the major care providers. This may also suggest the need to revise the undergraduate and graduate health science curricula to cover AMR and its interventions in Ethiopia (45).

Surgeons’ expertise lies in technical surgical procedures, necessitating the involvement of specialized care providers, such as infectious disease specialists, microbiologists, and clinical pharmacists, for optimal antibiotic management in surgery. This multidisciplinary approach can effectively augment the efforts of residents, medical interns, and primary service providers (44). A clear policy or procedure for sharing roles and responsibilities among anesthetists/anesthesiologists and nurses who are accountable for peri-operative antibiotic management is also key. These measures will assist in optimizing antibiotic administration, facilitating continuity of care, and helping to overcome commutation gaps observed in this study (46).

The inappropriate practice of SAP also stems from two key barriers: the absence of institutional policies or guidelines informed by local AMR data and the shortage of essential first-line drugs. The lack of binding local policies and guidelines provides an easy loophole for surgeons to prescribe broad-spectrum antimicrobials in default, even when narrower-spectrum agents are more appropriate.

Furthermore, the shortage of essential antimicrobials such as cefazolin, the first-line SAP, remains a critical issue despite its inclusion in the essential medicine list and the guidelines (17). This highlights the need for a comprehensive approach that addresses both supply chain issues and prescribing practices to ensure the availability and appropriate use of essential antimicrobials for SAP (17) (47, 48). In response to this study and other evidence of SAP malpractice, the Ministry of Health, in collaboration with donors, initiated an antimicrobial stewardship program across selected facilities (49). This report corroborates the findings of our study, demonstrating positive changes, though significant work remains.

Patient engagement in surgical infection-related care is variable and generally low worldwide (50). However, this aspect was not explored in our study due to factors such as limited literacy levels, including poor medical literacy, and a cultural preference for physician-directed decision-making (51).

When designing quality improvement initiatives, it is important to prioritize the prevailing practice and their inherent complexities (52). Involving seniors and recognizing their influence within the existing clinical environment is a key augmentation strategy to ensure the successful implementation of initiatives targeting appropriate SAP use (24, 26). Moreover, piloting and implementing antimicrobial stewardship (AMS) interventions followed by compliance audits are essential steps. Providing feedback and positive reinforcement of best practices by leadership is pivotal for achieving sustainable improvement (Table 4).

## Limitation

The study has the following limitations: It is a single-center study, which limits our ability to generalize the findings to other settings. The sampling technique may have also affected the results. We did not include infectious disease physicians, microbiologists, and pharmacists’ perspectives. Focus group discussions and quantitative surveys with adequate sample sizes could have provided additional insights into the complexities of antibiotic decision-making processes. The ward round was conducted during a period when medical interns were on break and COVID-19 restrictions were in place. The study may have also overlooked some critical data pertaining to external factors that could influence antibiotic decision-making, such as the impact of local antibiotic resistance patterns. While data triangulation, employing sequential data collection and analysis techniques, helped minimize bias, the lead researcher’s prior knowledge could have inadvertently introduced undetected biases. Despite these limitations and weaknesses identified, the study offers valuable insights into the current state of SAP practice in this hospital. The findings can guide future research and interventions aimed at improving SAP implementation and effectiveness, ultimately enhancing patient care and safety.

## Conclusion

The deeply ingrained customs within the clinical service and communication system significantly influence SAP decision-making and practices, often overriding evidence-based recommendations. This adherence to non-evidence-based practices, further exacerbated by inconsistencies in communication and documentation, shortage of drugs, and the absence of clear facility guidelines or policies, results in suboptimal SAP implementation. Engaging with SAP care services could optimize decision-making, communication, and antimicrobial usage, potentially reducing SSI complications. Implementing AMS practices could also effectively optimize SAP use in the surgical wards of TASH, further mitigating SSI risks.

## Data availability statement

The raw data supporting the conclusions of this article will be made available by the authors, without undue reservation.

## Ethics statement

The studies involving humans were approved by Ethical review board of the School of Pharmacy, College of Health Sciences, Addis Ababa Uni-versity. The studies were conducted in accordance with the local legislation and institutional requirements. Written informed consent for participation in this study was provided by the participants’ legal guardians/next of kin.

## Author contributions

GA, GTT, GAM, EG, and WA: conceptualization. GA: funding acquisition. AB, GA, and HM: data collection. GA, GTT, and WA: project administration. GA, GTT, and AB: data analysis and writing of the first draft. GA, GTT, WS, YD, GAM, AM, WG, HT, AA, EG, and WA: data curation. All authors reviewed, edited, and approved the final manuscript version.
