# Supplementary material for: Etiquette of the antibiotic decision-making process for surgical prophylaxis in Ethiopia: a triangulated ethnographic study
Source: Front Public Health. 2023 Dec 18;11:1251692. doi: 10.3389/fpubh.2023.1251692 (PMC10773818; doi:10.3389/fpubh.2023.1251692)
Supplement: Supplementary file 1 [file Data_Sheet_1.zip › Data Sheet 1/Annex 1_COREQ checklist.pdf]

Annex 1: Consolidated criteria for reporting qualitative research guidelines

The reporting of qualitative data collection and analysis methods was assessed for its adherence to the Consolidated Criteria for Reporting Qualitative Research (COREQ) checklist, a 32-item guideline for interviews and focus groups. The checklist is provided below, along with a corresponding column indicating where evidence for each criterion can be found in the study or providing justification for non-compliance.

(Source: https://academic.oup.com/intqhc/article/19/6/349/1791966 (Tong et al., 2007))

Number	Item	Guide questions/description	Cross-reference where included in report
**Domain 1: research team and reflexivity**			
**Personal characteristics**			
1.	Interviewer/facilitator	Which author/s conducted the interview or focus group?	Included in the *Study method* and see the researcher biographies in Annex 2)
2.	Credentials	What were the researcher's credentials? e.g. PhD, MD	Included in the researcher biographies in Annex 2)
3.	Occupation	What was their occupation at the time of the study?	Included in the researcher biographies in Annex 2)
4.	Gender	Was the researcher male or female?	Included in the researcher biographies in Annex 2)
5.	Experience and training	What experience or training did the researcher have?	Included in the researcher biographies in Annex 2)
**Relationship with participants**			
6.	Relationship established	Was a relationship established prior to study commencement?	Included in Annex 3
7.	Participant knowledge of the interviewer	What did the participants know about the researcher? *e.g. personal goals, reasons for doing the research*	Included in Annex 3
8.	Interviewer characteristics	What characteristics were reported about the interviewer/facilitator? *e.g. bias,*	Included in Annex 3

Number	Item	Guide questions/description	Cross-reference where included in report
		assumptions, reasons and interests in the research topic	
**Domain 2: study design**			
**Theoretical framework**			
9.	Methodological orientation and theory	What methodological orientation was stated to underpin the study? *e.g. grounded theory, discourse analysis, ethnography, phenomenology, content analysis*	Included in the *Study method* and *discussion* section.
**Participant selection**			
10.	Sampling	How were participants selected? *e.g. purposive, convenience, consecutive, snowball*	Included in the *Study method (*under the *Interview)*
11.	Method of approach	How were participants approached? *e.g. face to face, telephone, mail, e-mail*	Included in the *Study method (*see under the *Interview* subtopic*)*
12.	Sample size	How many participants were in the study?	Included in the *Study method*
13.	Non-participation	How many people refused to participate or dropped out? Reasons?	Included in the *Study method (*see under the *Interview* subtopic*)*
**Setting**			
14.	Setting of data collection	Where was the data collected? *e.g. home, clinic, workplace*	Included in the *Study method (*see under the *Interview* subtopic*)*
15.	Presence of non-participants	Was anyone else present besides the participants and researchers?	Included in the *Study method (*see under the *Interview* subtopic*)*
16.	Description of sample	What are the important characteristics of the sample? *e.g. demographic data, date*	Included in the *Study method (*see under the *Interview* subtopic*)*
**Data collection**			
17.	Interview guide	Were questions, prompts, guides provided by the authors? Was it pilot tested?	Yes, included in the *Study method*
18.	Repeat interviews	Were repeat interviews carried out? If yes, how many?	Repeat interviews were not conducted and are not reported

Number	Item	Guide questions/description	Cross-reference where included in report
19.	Audio/visual recording	Did the research use audio or visual recording to collect the data?	Included in the *Study method (*see under the *Interview* subtopic*)*
20.	Field notes	Were field notes made during and/or after the interview or focus group?	Included in the *Study method*
21.	Duration	What was the duration of the interviews or focus group?	Included in the *Study method (*see under the *Interview* subtopic*)*
22.	Data saturation	Was data saturation discussed?	Included in the *Study method (*see under the *Interview* subtopic*)*
23.	Transcripts returned	Were transcripts returned to participants for comment and/or correction?	Transcripts were not returned to participants and are not reported
**Domain 3: analysis and findings**			
**Data analysis**			
24.	Number of data coders	How many data coders coded the data?	Included in the *Study method (*see *Data rigor and analysis)*
25.	Description of the coding tree	Did authors provide a description of the coding tree?	Not reported
26.	Derivation of themes	Were themes identified in advance or derived from the data?	Derived from the data using a deductive method
27.	Software	What software, if applicable, was used to manage the data?	No – described in the *Study method (*see *Data rigor and analysis)*
28.	Participant checking	Did participants provide feedback on the findings?	Feedback was not received from participants and are not reported
**Reporting**			
29.	Quotations presented	Were participant quotations presented to illustrate the themes/findings? Was each quotation identified? *e.g. participant number*	Yes – demonstrated throughout the findings

Number	Item	Guide questions/description	Cross-reference where included in report
30.	Data and findings consistent	Was there consistency between the data presented and the findings?	Yes – demonstrated throughout the findings
31.	Clarity of major themes	Were major themes clearly presented in the findings?	Yes – demonstrated throughout the findings. Themes with handful descriptions and salient transcripts have been included (Table 3)
32.	Clarity of minor themes	Is there a description of diverse cases or discussion of minor themes?	Yes – demonstrated throughout the findings. Themes with handful descriptions and salient transcripts have been included
