# Supplementary material for: Etiquette of the antibiotic decision-making process for surgical prophylaxis in Ethiopia: a triangulated ethnographic study
Source: Front Public Health. 2023 Dec 18;11:1251692. doi: 10.3389/fpubh.2023.1251692 (PMC10773818; doi:10.3389/fpubh.2023.1251692)
Supplement: Supplementary file 1 [file Data_Sheet_1.zip › Data Sheet 1/Annex 2, Researchers demographics.pdf]

Annex 2: Qualitative research study profiles

GA and GTT, are clinical pharmacy lecturers and researchers with master's degrees, employed by the School of Pharmacy at Addis Ababa University both during the data collection and currently. GA has since joined the USAID Medicines and Pharmaceutical Services (USAID MTaPS) Program.

AB, a clinical pharmacy postgraduate student at the time of data collection and currently, and HM, a female clinical pharmacy postgraduate student and currently a researcher, were both employed as lecturers and researchers by Addis Ababa University both during and after the data collection process.

WS and YD were antimicrobial resistance case team members at the Ministry of Health during data collection (YD is now affiliated with the Afro World Health Organization). WG and HT are pharmacists with master's degrees who were employed by the USAID MTaPS Program both at the time of data collection and currently. EG is a behavioral health specialist with a PhD and is employed by the School of Public Health at Addis Ababa University. GAM is a physician with an MD degree who was a neurosurgery resident at Tikur Anbessa Specialized Hospital affiliated with Addis Ababa University during data collection and currently works at Yekatit 12 Hospital in Addis Ababa. AM is an internist, AA is a surgeon, and WA is an infectious disease specialist. All three physicians hold MD and PhD degrees and are clinicians and academicians employed by Tikur Anbessa Specialized Hospital, Addis Ababa University.

AB and HM were responsible for data collection under the supervision of GA. They also participated in data transcription, and coding. Their limited prior exposure to the field might have influenced the coding process, but their analytical insights largely aligned with GA's.

WS, YD, WG, and HT possess prior experience in the field and some familiarity with the literature. GA and GTT have expertise in quantitative research but limited experience with qualitative methods. They were involved in data analysis, with GA leading the process as the primary researcher. GA supervised data collection and conducted the analysis. Despite his limited qualitative research background, he underwent a four-day training course six months before the project data collection. GA and data collectors also took two days trainings on research methods with a focus on Ethnographic study prior to the data collection. Both trainings

were delivered by EG. GA's prior publications in the field of surgical antibiotic use and exposure to the relevant literature might have influenced his data interpretation and analysis, but not the raw data itself.

AM, AA, and WA have extensive experience in the field and its literature. GAM has no experiences in qualitative research. GAM, AA, and WA supported the data collectors by clarifying quires. EG is a qualitative research expert with a rich background in literature review. He provided guidance during data analysis. The initial analytical draft prepared by GA was discussed with GTT and the data collectors, and any discrepancies were resolved through consultations with EG. We have no reason to believe that any of the researchers' personal experiences or life histories had a significant impact on their approaches to data collection and analysis.
