# Supplementary material for: Etiquette of the antibiotic decision-making process for surgical prophylaxis in Ethiopia: a triangulated ethnographic study
Source: Front Public Health. 2023 Dec 18;11:1251692. doi: 10.3389/fpubh.2023.1251692 (PMC10773818; doi:10.3389/fpubh.2023.1251692)
Supplement: Supplementary file 1 [file Data_Sheet_1.zip › Data Sheet 1/Annex 3, Participant prior relationship.pdf]

Annex 3. Qualitative research study prior relationship/contact with research participants

AB, HM, GTT, and GA have been colleagues since before the data collection.

AM, AA, and WA have a history of working together.

GA has previously interacted with WS, YD, WG, and HT, who also have established work relationships among themselves in the context of various national antimicrobial resistance initiatives.

GA has had prior one-on-one interactions with GAM, AM, and WA in the workplace.

GA, AM, and WA collaborated on revising the infectious disease section (including surgical antibiotic prophylaxis) of the national guidelines.

GA and EG met for the first time during the qualitative data training six months before this research project's conception.

GA established connections with AA for the first time in relation to this research.

Participants in the ward round and clinical practice observations were not provided with information about the data collectors and researchers. Participants in the interview were provided with an overview of the research and did not include any personal details about the researchers or their research interests. As a result, participants were not informed of any personal information about the researchers before the interviews. However, some participants may have been known with the researchers from their professional interactions or may have also had the opportunity to read about their research interests online. While this potential influence was not explicitly addressed in the observation and interviews, there is a possibility that participants' prior knowledge of the researchers' interests may have influenced their responses.
